# Does white matter structure or hippocampal volume mediate associations between cortisol and cognitive ageing?

**DOI:** 10.1016/j.psyneuen.2015.08.005

**Published:** 2015-12

**Authors:** Simon R. Cox, Sarah E. MacPherson, Karen J. Ferguson, Natalie A. Royle, Susana Muñoz Maniega, Maria del C. Valdés Hernández, Mark E. Bastin, Alasdair M.J. MacLullich, Joanna M. Wardlaw, Ian J. Deary

**Affiliations:** a Centre for Cognitive Ageing and Cognitive Epidemiology, University of Edinburgh, UK; b Department of Psychology, University of Edinburgh, UK; c Brain Research Imaging Centre, Neuroimaging Sciences, University of Edinburgh, UK; d Edinburgh Delirium Research Group, Geriatric Medicine, University of Edinburgh, UK; e Scottish Imaging Network, A Platform for Scientific Excellence (SINAPSE) Collaboration, Edinburgh, UK

**Keywords:** Cortisol, White matter, Hippocampus, Cognitive change, Ageing

## Abstract

Elevated glucocorticoid (GC) levels putatively damage specific brain regions, which in turn may accelerate cognitive ageing. However, many studies are cross-sectional or have relatively short follow-up periods, making it difficult to relate GCs directly to changes in cognitive ability with increasing age. Moreover, studies combining endocrine, MRI and cognitive variables are scarce, measurement methods vary considerably, and formal tests of the underlying causal hypothesis (cortisol → brain → cognition) are absent. In this study, 90 men, aged 73 years, provided measures of fluid intelligence, processing speed and memory, diurnal and reactive salivary cortisol and two measures of white matter (WM) structure (WM hyperintensity volume from structural MRI and mean diffusivity averaged across 12 major tracts from diffusion tensor MRI), hippocampal volume, and also cognitive ability at age 11. We tested whether negative relationships between cognitive ageing differences (over more than 60 years) and salivary cortisol were significantly mediated by WM and hippocampal volume. Significant associations between reactive cortisol at 73 and cognitive ageing differences between 11 and 73 (*r *= −.28 to −.36, *p *< .05) were partially mediated by both WM structural measures, but not hippocampal volume. Cortisol-WM relationships were modest, as was the degree to which WM structure attenuated cortisol–cognition associations (<15%). These data support the hypothesis that GCs contribute to cognitive ageing differences from childhood to the early 70s, partly via brain WM structure.

## Highlights

• 90 older men provided salivary cortisol, brain MRI and cognitive measures.
• Reactive but not diurnal cortisol was related to cognitive change over >60 years.
• White matter integrity but not hippocampal volume mediated this relationship.
• Cortisol may contribute to lifetime cognitive ageing via white matter structure.

## 1 Introduction

Exposure to elevated glucocorticoid (GC) levels is hypothesised to have deleterious effects on brain structure (or specific regions or tissue types thereof), which negatively affect cognitive function (Landfield et al., 2007, Sapolsky et al., 1986). GCs (cortisol in humans) exhibit a complex diurnal pattern and also a phasic response to psychological and physical challenge. Increasing age is associated with altered diurnal and reactive profiles of GCs (cortisol in humans; Heaney et al., 2010, Otte et al., 2005) as well as average declines in brain structure and cognitive function. Several studies using moderate-to-large sample sizes suggest that impairments in domains of processing speed, memory and global cognition are associated with higher diurnal and reactive cortisol measures in older adults (97 ≤ *n *≤ 1154; Comijs et al., 2010, Gerritsen et al., 2009, Kuningas et al., 2007, Lee et al., 2007, MacLullich et al., 2005). Separately, other studies suggest that cognitive ageing may be mediated via brain structure (discussed below), yet, studies with all three types of data—cortisol, brain structure, and cognition—are rare, and no formal tests of mediation have been undertaken to directly test this central three-component hypothesis. Synthesis of findings is hampered by the variety of cognitive measures employed, and diurnal and reactive cortisol (e.g. in response to a psychological stressor) are seldom reported together. The absence of information about prior cognitive ability in the majority of previous studies also makes cross-sectional analyses of GCs, brain and behaviour difficult to interpret because the overarching causal hypothesis posits a change of cognition over time, dependent upon GC levels.

With respect to possible mediators of cortisol-cognition relationships, the hippocampus is one putative neural substrate of these effects. The initial hypothesis of GC-induced hippocampal cell death has been moderated by evidence of reversible dendritic atrophy in the rodent hippocampus (Conrad, 2009, McEwen and Gianaros, 2010) and GC-induced impairments in memory tasks (Dachir et al., 1993, Sousa et al., 2000). However, evidence of significant associations between cortisol levels and hippocampal volume among healthy older humans is limited. Lupien et al. (1998) reported that 11 individuals with increasing 24 h cortisol over 5 years had decreasing hippocampal volumes and concomitant declines in memory. Subsequent studies in larger samples report no significant relationship between cortisol and hippocampal measures in non-pathological ageing (Coluccia et al., 2008, Gold et al., 2005, Kremen et al., 2010a, MacLullich et al., 2005, MacLullich et al., 2006, MacLullich et al., 2012).

There is also evidence that the brain’s white matter (WM) may be susceptible to elevated GC effects. WM is significant because of its relationship with cognitive ability (Filley, 2010, Penke et al., 2012) and because it supports efficient information transfer between brain regions involved in supra-HPA axis regulation (Ulrich-Lai and Herman, 2009). There is evidence that elevated GCs alter cerebral white matter microstructure. In rodents, administering a GC receptor agonist following brain lesion impaired axonal sprouting in a dose-dependent manner when compared to controls (Scheff and Cotman, 1982, Scheff and DeKosky, 1983). Elevated GCs and stress inhibit the proliferation of astrocytes and oligodendrocytes (Alonso, 2000, Miyata et al., 2011, Rajkowska and Miguel-Hidalgo, 2007), suggesting that GCs may play a role in hindering oligodendrocyte-mediated axonal remyelination. In humans, Cushings syndrome patients reportedly exhibit widespread loss of white matter integrity and predominant demyelination (Pires et al., 2015), and these differences persist many years after remission (van der Werff et al., 2014). In healthy older adults, higher evening cortisol was associated with lower estimates of periventricular WM structure (Macritchie et al., 2013). In the same older males as in the present study, we reported that elevated reactive cortisol at the start or end of cognitive testing was associated with lower WM structural estimates (Cox et al., 2015). In the current study, we therefore, expand the analyses to report novel associations between cortisol and cognitive ability, and also novel associations between cortisol and hippocampal volume. We then use mediation analysis to test the hypothesis that these cortisol–brain associations might have ramifications for cognitive ageing differences.

In summary, cross-sectional bivariate analyses have been the mainstay of studies which test the hypothesis that elevated cortisol has deleterious effects on the brain and cognitive functioning. Yet there remain no attempts to test whether cortisol–cognition relationships are mediated by poorer brain structure. This may be due to a lack of studies that collect all three requisite domains of data, or to the file-drawer effect in previous studies with all three types of data (i.e. no significant 3-way associations—required for mediation analysis—were found). This, in turn, may be due to the focus on hippocampal measures whereas, in fact, other brain indices (such as WM measures) might be just as – or more – pertinent to cognitive performance among healthy older individuals. Finally, inferring whether cortisol affects cognitive decline in old age requires knowledge of how a subject is performing now, relative to their prior ability. This information is absent in many existing GC studies which use cross-sectional cognitive indices, and longitudinal cognitive data are few, spanning only 6 years or less. In the current study, we examine a group of 90 older males who have provided both diurnal and reactive salivary cortisol measures, hippocampal volume and measures of white matter structure measured at age 73 years, and cognitive ability scores obtained at ages 11 and 73 years. The association between childhood and older-age cognitive ability measured in this way typically ranges between .60 to .70 (reviewed in Deary, 2014), indicating that intelligence between youth and old age is remarkably stable throughout the lifecourse. Importantly, however, the association is not perfect, allowing investigations into which factors might be related to cognitive ageing differences. In this instance, these data afford a rare opportunity to test the hypothesis that elevated cortisol levels are associated with negative cognitive change (over more than 60 years) via negative relationships between cortisol and hippocampal volume or WM structure.

## 2 Material and methods

### 2.1 Participants

The participants were 90 older community-dwelling males from the Lothian Birth Cohort 1936 (LBC1936). The members of this cohort were born in 1936 and sat a valid IQ-type test (Moray House Test No. 12; MHT) at school in Scotland in June 1947 at an average age of 11 years. At around 70 years of age, 1091 surviving, healthy, community-dwelling residents in the Edinburgh and Lothians area, most of whom had taken this test in 1947, were recruited as the LBC1936 and underwent a series of cognitive and physical tests (Deary et al., 2007). Three years later, 866 returned for a second follow-up wave of cognitive testing and an MRI brain scan (Deary et al., 2012, Wardlaw et al., 2011).

From this second wave of LBC1936 testing, male participants were selected on the following criteria: a score of 24 out of 30 or greater on the MMSE (Folstein et al., 1975), a score less than 11 out of 21 on the depression facet of the Hospital Anxiety and Depression Scale (Snaith, 2003), a complete MRI scan, not taking any antidepressant or glucocorticoid medication, free from diagnosis of neurodegenerative disorders and no history of serious neurological event (brain scans were examined by a consultant neuroradiologist; JMW). Of 118 potential participants, 90 agreed to take part. These participants were of mean age 73.10 (SD 0.40) years when they sat a battery of cognitive tests (see Section 2.3). At MRI scanning, they had a mean age of 73.30 (SD 0.37) years. They were invited to attend an appointment in a novel (for them) location (Department of Psychology, University of Edinburgh) to attend a cognitive testing session during which salivary cortisol was collected at the start and end of the testing session; diurnal salivary cortisol was sampled on waking and at 10pm on a separate weekday (see Section 2.2). Salivary cortisol sampling took place just over one year after MRI acquisition (mean 431.42 days, SD 103.62). Written informed consent was obtained from each participant and the study was conducted in compliance with departmental guidelines on participant testing and the Declaration of Helsinki. Ethical approval was gained from NHS Lothian Research Ethics Committee (NREC:07/MRE10/58) and the Philosophy, Psychology and Language Sciences Research Ethics Committee at the University of Edinburgh.

### 2.2 Cortisol

Salivette devices (Sarstedt, Numbrecht, Germany) were used and samples stored at −80 °C following collection. Assays were carried out by Dresden LabService GmbH in accordance with a Material Transfer Agreement. All measures are reported in nmol/l. Reactive cortisol was measured at the start and end of a cognitive testing appointment (referred to as START and END, respectively; Cox et al., 2015). Efforts were made to normalise the testing time for each participant, which took take place three hours following waking on that day (Mean = 3 h 16 mins, SD = 51 mins). Diurnal measures were taken on a separate weekday on natural waking and 10pm at night (WAKING and EVENING, respectively). REACTIVE SLOPE and DIURNAL SLOPE were calculated by subtracting the earlier (WAKING or START) from the later measure (EVENING or END). Thus, a decreasing slope is denoted by a negative value.

### 2.3 Cognitive tests

Age 11 cognitive ability was measured using participants’ total MHT score, administered in June 1947 as part of the Scottish Mental Survey. The test included items on reasoning, word classification and other verbal, spatial and arithmetical items with a 45 min time limit allowed. The total score was concurrently validated against the Terman–Merrill revision of the Binet Scales (Scottish Council for Research in Education, 1932).

At age 73, three cognitive domains were measured from the first unrotated component of three separate principal component analyses (PCAs), reflecting prior demonstrations of fluid intelligence (or *g*_f_) as hierarchically superordinate and non-orthogonal to the cognitive domains of processing speed and memory, each of which are affected by increasing age (e.g. Deary, 2001, Salthouse, 2004). Fluid intelligence (or *g*_f_) was derived from Matrix Reasoning, Block Design, Digit Span Backward and Letter–Number Sequencing subtests from the Wechsler Adult Intelligence Scale, 3rd UK Edition (WAIS-IIIUK; Wechsler, 1998a), accounting for 55.79% of the variance. Processing speed used the Digit–Symbol Coding and Symbol Search tests from the WAIS III-UK, two measures from a bespoke reaction time box (Simple and Four-Choice reaction time; Deary et al., 2001) and one visual inspection time task on a computer (Deary et al., 2004), and accounted for 52.47% of the variance. Memory was measured using Logical Memory, Verbal Paired Associates, Letter–Number Sequencing, Spatial Span and Backward Digit Span subtests from the Wechsler Memory Scale 3rd UK Edition (WMS III-UK; Wechsler, 1998b), accounting for 53.79% of the variance.

These cognitive domain scores at age 73 were corrected for age 11 cognitive ability using linear regression in which the residual value represents the deviation in age 73 cognitive ability (*g*_f_, Speed, Memory) from that predicted by cognitive ability at age 11. This yielded the residual scores (*g*_f_R, SpeedR and MemoryR), where a negative value indicates a decline from initial ability in cognition relative to others in the sample.

### 2.4 MRI acquisition

All MRI data were obtained from a GE Signa Horizon HDxt 1.5 T clinical scanner (General Electric, Milwaukee, WI, USA) using a self-shielding gradient set with maximum gradient strength of 33 m/Tm, and an 8-channel phased-array head coil. The full MRI protocol has been described in detail elsewhere (Wardlaw et al., 2011). Briefly, image acquisition comprised whole-brain structural T_2_-, T_2_*- and fluid-attenuated inversion recovery (FLAIR)-weighted axial scans, and a high-resolution 3D T_1_-weighted (T_1_W) volume sequence (voxel-dimensions 1 × 1 × 1.3 mm), acquired in the coronal plane, along the hippocampal long-axis. The DT-MRI protocol employed single-shot spin-echo echo-planar diffusion-weighted volumes (*b *= 1000 s mm^−2^) acquired in 64 non-collinear directions, along with seven T_2_-weighted images (*b* = 0 s mm^−2^). Seventy-two contiguous axial slices of 2 mm thickness were acquired with a field of view of 256 × 256 mm and matrix size of 128 × 128, giving a resolution of 2 × 2 × 2 mm^3^. Repetition and echo times were 16.5 s and 95.5 ms, respectively. Total image acquisition took approximately 70 min.

### 2.5 MRI analysis

Hippocampal volumes were initially segmented automatically using FSL FIRST (T_1_W registered to an age-appropriate template; Farrell et al., 2009) before visual assessment and manual editing by one of the authors (NAR). The method was applied to all LBC1936 participants (see Aribisala et al., 2014 for example) with excellent intra-rater reliability (ICC = .98 in a sample of 103; an example of hippocampal segmentation is provided in Supplementary Fig. 1). Automated segmentation of the hippocampus failed in one case, leaving 89 participants with available hippocampal volumes (mm^3^) corrected for intracranial volume. Intracranial volume, measured by one of the authors (NAR), included all structures and CSF inside the dura. The lower limit was the axial slice immediately inferior to the inferior limit of the cerebellar tonsils on or above the superior tip of the odontoid process (Valdés Hernández et al., 2012, Wardlaw et al., 2011).

Two complementary methods to measure WM structure were employed. The first allows an estimate of WM structure by indexing water molecule diffusion along major axonal fibre bundles using DT-MRI and tractography. The second quantifies the total volume of WMH—a common feature of the ageing brain, visible on T_2_- and FLAIR-weighted MRI.

Probabilistic neighbourhood tractography (PNT) was used to measure MD in twelve major long-range WM fasciculi: bilateral anterior thalamic radiation, cingulum, uncinate, arcuate and inferior longitudinal fasciculi, and the genu and splenium of the corpus callosum. Initial pre-processing involved extracting the brain, removing bulk participant motion and eddy current-induced distortions (by registering the component EPI data to the first T2-weighted volume), before water diffusion tensor parameters were estimated using FSL tools (FMRIB, Oxford UK; www.fmrib.ox.ac.uk). Brain connectivity data were created using the BEDPOSTX/ProbTrackX algorithm with a two-fibre model and 5000 streamlines to reconstruct tracts of interest. PNT as implemented in the TractoR package (http://www.tractor-mri.org.uk; Clayden et al., 2011) was used to identify the tracts of interest from these connectivity data. For each of the 12 fasciculi-of-interest, a seed point was transferred from the centre of the reference tract (in standard space, derived from a digital white-matter atlas; Bastin et al., 2010) into the native space of the brain to-be-analyzed. Around that native space central seed point, a 7 × 7 × 7 volume is created from which single seed point tractography is initiated. For each of the 343 voxels, the resulting ‘candidate’ tracts are compared against the reference in terms of their length and shape. The one with the closest match to the reference is then chosen; tract segmentation was further improved by using the topological tract model to reject false positive connections (Clayden et al., 2011). For each tract in each participant, the seed point that produced the best match to the reference tract was used to create the tractography mask from which the tract-averaged MD values were calculated. The resultant values for each tract were entered into a PCA and extracted the first unrotated component to derive a tract-averaged score of general mean diffusivity (*g*_MD_) explaining 51.39% of the variance (Cox et al., 2015*;* Penke et al., 2012). A measure of general FA–similarly derived–is excluded from mediation analysis because we previously reported no significant associations with any cortisol measures in this sample.

WMH were identified as punctate or diffuse areas that were hyperintense compared to normal-appearing white and grey matter on T_2_-weighted and FLAIR images. Their volume was initially quantified using the automated multi-spectral data fusion method MCMxxVI (Valdés Hernández et al., 2010). Each segmentation was then visually inspected and manually corrected for false-positives and omissions.

### 2.6 Statistical analysis

All data were examined for extreme values (±3 SD) and normality of distribution prior to knowledge of their relationships with other variables of interest. Extreme brain imaging values were removed if they clearly represented measurement error or partial volume effects. Those that were found to be accurate were winsorised in order to preserve meaningful data but minimise their disproportionate effect on parametric statistics. A single marginal outlier was identified in both left and right hippocampi; these were winsorised following examination of object maps by one of the authors (NAR). Following examination of tract segmentation quality (by SMM), tracts affected by partial volume averaging of CSF signal or those which contained aberrant or truncated pathways that were not anatomically plausible representations of the fasciculi of interest were removed. Example of tracts deemed unacceptable are shown in Fig. 4 of Bastin et al. (2008). All outlying cortisol data points (12; i.e. 3% of the total 360 samples) were removed since there is a weaker correspondence between salivary and serum cortisol with increasing concentrations (Hellhammer et al., 2009). The outlying points were all higher than the 95th percentile of unpublished diurnal salivary cortisol data from up to 13,366 humans, collected at Dresden LabService GmbH, where the assays were undertaken (Clemens Kirschbaum, personal communication). A log transformation of cortisol measures at EVENING, START and END and a square root transformation of WMH volume normalised skewed distributions.

We then tested relationships between (1) cortisol levels and cognitive ageing differences, (2) MRI measures and cognitive ageing differences, and (3) cortisol and MRI measures. Two-tailed Pearson’s product-moment correlations (*r*) are reported throughout. Mediation analysis was then used to examine the hypothesis that poorer cognition is related to higher cortisol via the negative relationship between cortisol and MRI measures; in other words, brain structure significantly mediates the negative relationship between cortisol and cognition.

**Fig. 1:**
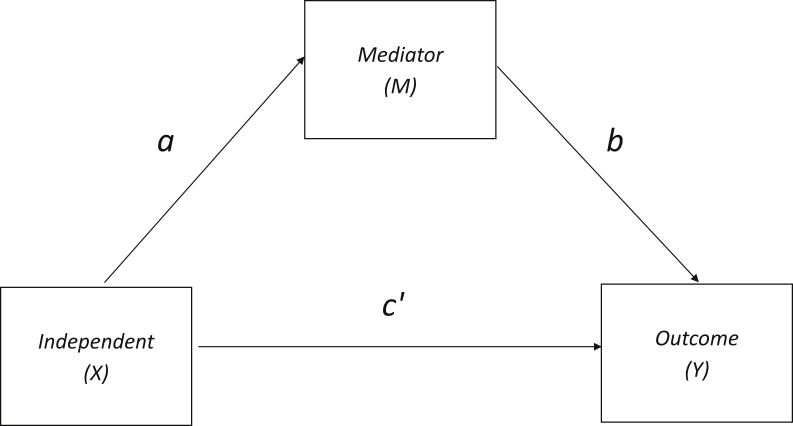
A schematic relationship in which an observed relationship between independent (*X*) and outcome (*Y*) variables is hypothesised to be explained by a mediator variable *M*. The direct effect of *X* on *M* is *a*, the effect of *M* on *Y* is *b*, and *c*’ denotes the *X → Y* path coefficient after controlling for *M*. Therefore the indirect effect of *X* on *Y* is the product of the *X *→ *M*, and *M → Y* paths, or *ab.* For the purposes of this study, *X* = cortisol, *M *= brain structure, *Y *= cognition.

The most commonly-used mediation approach was proposed by Baron and Kenny (1986) (Fig. 1). The causal steps strategy stipulates that mediation is present if the *X* *→ Y* relationship attenuates to non-significance when accounting for *M*, or that partial mediation is present when the magnitude of *X *→ *Y* is reduced after *M.* However, rather than concluding that mediation is present through observation, we used the INDIRECT macro in IBM SPSS 19 (Preacher and Hayes, 2008) to assess mediation effects (the difference between *X* *→ Y* and direct c’ effects) using 5000 bootstrapped samples. This offers increased power over other methods, such as the Sobel test, because it makes no assumptions about distribution normality and guards against instances where the change from significance to non-significance is accompanied by a negligible decrease in coefficient size (Type I error), or where a large change in coefficient size is accompanied by no ostensible change in significance (Type II error; Preacher and Hayes, 2004). Based on the clearly directional hypotheses, one-tailed tests of mediation were conducted (A. Hayes: http://www.afhayes.com/macrofaq.html). Mediation effects are present if the confidence interval span does not include zero (Preacher and Hayes, 2008).

## 3 Results

### 3.1 Descriptive statistics

Descriptive statistics of salivary cortisol measures and MRI variables are provided in Table 1. Measures of general WM mean diffusivity (*g*_MD_), general cognitive ability (*g*) and the domains of processing speed (Speed) and Memory were all normalised residuals with an approximate mean of 0 and a standard deviation of 1. As previously reported, participants’ cortisol profiles generally exhibited the expected pattern. WAKING levels were higher than in the EVENING, START levels were higher than at END, and the majority of participants’ START levels were higher or within 5 nmol/l of the value predicted by their linearly-modelled diurnal slope (Cox et al., 2015).

### 3.2 Cortisol and cognitive ageing differences

Consistent with prior reports, age 11 IQ was a significant predictor of *g* (*r = *.70, *p *< .001), speed (*r = *.45, *p *< .001) and memory (*r = *.70, *p *< .001) in older age. Correlations between reactive and diurnal cortisol and cognitive abilities (residualised for age 11 IQ) are shown in Table 2. Higher START and END cortisol was associated with poorer cognition across all measures (*r *= −.28 to −.36, *p *< .05). However, neither WAKING, EVENING, nor either slope measure (DIURNAL SLOPE or REACTIVE SLOPE) were significantly associated with measures of cognitive ageing differences. Given the highly significant correlation between START and END levels (*r* (84) = .45, *p *< .001), and both levels being associated with cognition, we used hierarchical linear regression to examine whether these cortisol measures both contributed uniquely to the variance in cognitive change. The result (Table 3) provides some evidence to support this. Both START and END levels were significant contributors to MemoryR when entered together (*R*^2^ = 18%). However, END only showed a non-significant trend when START was also included in the SpeedR model (*R*^2^ = 17%) and START levels did not contribute significantly when END was entered into the *g*_f_R model (*R*^2^ = 13%).

### 3.3 Cortisol and brain imaging variables

Correlations between cortisol levels and brain imaging measures are shown in Table 2. There were no significant relationships between any cortisol measure and hippocampal volume. Aside from an association between higher EVENING and higher *g*_MD_, there were no significant relationships between diurnal cortisol and any brain imaging variables. However, those with higher START cortisol levels had poorer WM structure (higher *g*_MD_ or a greater WMH volume). Higher END levels were also significantly associated with greater WMH volume. Contrary to expectations, a more steeply negative REACTIVE SLOPE (denoting a greater decrease in cortisol levels between START and END) was associated with lower (i.e. better) *g*_MD_.

### 3.4 Brain imaging variables and cognitive ageing differences

Correlations between brain imaging and cognitive variables (Table 2) were in the expected direction in every case (less cognitive change with larger hippocampal volume, lower WMH and lower *g*_MD_). These were significant in every case (magnitudes raged from .23 to .35, *p *< .05) except for the relationship between *g*_f_R and WMH. Though REACTIVE SLOPE was related to *g*_MD_ (Section 3.3), it was not relevant to cognitive ageing differences.

### 3.5 Mediation analysis

Bootstrapping statistics were conducted on candidates for mediation analysis, selected based on the presence of three-way associations (cortisol–brain–cognition). Given the results of the bivariate analyses above, mediation analyses involved START or END cortisol measures as the independent (*X*) variable, *g*_MD_ or WMH as candidate mediators (*M*), and all three cognitive measures as outcome (*Y*) variables. Results of the analyses are shown in Table 4. They indicate significant mediation of the relationship between cortisol and cognitive ageing differences by WM measures in all but two instances (START → WMH *→ g*_f_R and END → WMH → *g*_f_R).

## 4 Discussion

In this study we found that cortisol levels measured at the start and end of a cognitive test battery among older men were positively associated with a relative decline in general cognitive ability, processing speed and memory functioning. Poorer WM structure and smaller hippocampi were generally related to a greater relative decline in cognition. However, only measures of WM—not hippocampal volume—were associated with elevated START and END levels of cortisol. When formally tested using a bootstrapping method, neuroradiologically complementary measures of WM microstructure (*g*_MD_) and macrostructure (WMH) showed significant partial mediation of cortisol–cognition relationships in 7 out of 9 candidate pathways. Taken together, these preliminary and exploratory data support the hypothesis that reactive cortisol measures are pertinent to life-course changes in cognitive ability, partially through cortisol’s detrimental effects on WM structure, but not via hippocampal volume.

We did not find a significant association between diurnal cortisol values and older age cognition, in contrast with some – but by no means all – cross-sectional studies in older adults. Some longitudinal studies do not implicate diurnal measures in cognitive change (Seeman et al., 1997, MacLullich et al., 2012, Singh-Manoux et al., 2014; but see also Li et al., 2006, Beluche et al., 2010). One could argue that the short follow up period in these studies (≤6 years) may be too short to detect small effects reliably, but we find no indication of these effects on cognitive change over a period of 60 years. Instead, we found that relationships with cognitive ageing differences were specific to salivary cortisol at the START and END of cognitive testing. Moreover, these two levels were only moderately correlated (.45), suggesting that not all individuals with high START levels necessarily have high END levels. The hierarchical linear regression further suggests that both levels provide partially non-overlapping information about cognition (and both these cortisol measures and indices of cognition are related to white matter). Our findings accord with other reported associations between greater reactive cortisol and poorer cognition in older age (MacLullich et al., 2005, Lee et al., 2007) and those linking elevated cortisol or stress reactivity with poorer WM indices (Scheff and Cotman, 1982, Scheff and DeKosky, 1983).

The primary mechanism through which cortisol has been linked with reduced white matter integrity is via the inhibition of repair mechanisms (Alonso, 2000, Miyata et al., 2011, Pires et al., 2015, Rajkowska and Miguel-Hidalgo, 2007, Scheff and Cotman, 1982, Scheff and DeKosky, 1983, van der Werff et al., 2014). As such, individual differences in HPA axis response to cognitive stressors may interfere with white matter repair following accumulated insult over the lifecourse, resulting in poorer integrity, impaired information transfer between regions with increasing age, thus influencing cognitive ageing differences. However, this study precludes direct testing of cellular mechanisms, and little is currently known about the test-retest reliability of reactive salivary measures (i.e. whether they measure a trait of biological response to challenge), which could benefit from future investigation.

Our data are not consistent with a role for the hippocampus in cortisol-related cognitive decline. Rodent models and early human research supported the role of elevated GCs in ageing as a mechanism of hippocampal damage and memory impairment (Lupien et al., 1998, Sapolsky et al., 1986). However, the present study is one of an increasing number to have found no association between hippocampal volume and cortisol in older humans (Coluccia et al., 2008, Gold et al., 2005, Kremen et al., 2010a, MacLullich et al., 2005, MacLullich et al., 2006, MacLullich et al., 2012), suggesting that hippocampal volume may not be the primary mediator of this association in healthy ageing. Nevertheless, this does not exclude the possibility that the hippocampus is a target of deleterious GC-related change. Exposure to clinically high levels in Cushing’s disease results in reductions of hippocampal volume (reviewed in Patil et al., 2007) but others report volumetric reduction only in the head of the hippocampus (Toffanin et al., 2011 though the degree to which hippocampal atrophy in Cushing’s is independent of global cerebral atrophy is unclear). The more subtle effects of non-pathological cortisol levels (such as those in healthy ageing) on the hippocampus could be subregional and therefore not detectable by gross volumetry. Some rodent studies have identified putative effects of GC exposure in areas CA1 and CA3 only (reviewed in McEwen, 2007). Although both mineralocorticoid and glucocorticoid receptors (GR) are found in the hippocampus (De Kloet et al., 1998), GR may be less preferentially expressed in primates and humans than rodents in this region (Sanchéz et al., 2000), potentially accounting for discrepancies between human and rodent studies. Thus, taken with other evidence, this study suggests that the cortisol levels exhibited by relatively healthy older adult humans may be insufficient to cause detectable changes in hippocampal volume.

We also note that WM measures only partially attenuated the relationship between cortisol and cognitive change in our data. What might account for the remainder of the variance? One possibility is that cortisol is related to the integrity of other brain regions not reported. For example, the prefrontal cortex and amydgala have both been implicated in HPA axis regulation and are putative targets of cortisol-driven remodelling (e.g. Dedovic et al., 2009). The brain’s WM and the hippocampus may also be affected in ways that the current methods (diffusivity and volumetry) are unable to detect. MRI-derived measures of longitudinal relaxation time and magnetisation transfer ratio are promising alternative indices of cerebral integrity (both exhibit stronger associations with older-age cognition than volumetric indices in the hippocampus; Aribisala et al., 2014). It is also possible that the magnitude of cortisol–cognition mediation varies by white matter region or tract; a question for which the current study lacks the requisite statistical power. Thus, future studies could usefully examine the cognitive correlates of cortisol profiles in relation to subregional hippocampal volume, white matter tract–specific relationships, other candidate brain regions, employing multi-modal MRI analyses.

The present study has several limitations. The current measure of cognitive ageing differences from age 11 to 73 years is influenced by periods of both neurodevelopment and neurodegeneration, which the current study is unable to parse apart. We sampled salivary cortisol only once at each time point, though it has been recommended that at least two separate occasions are preferable for diurnal cortisol estimation (Kraemer et al., 2006). In addition, we cannot know the level of adherence to the diurnal sampling protocol. Though self-report from our participants and data from previous studies (Kraemer et al., 2006, Almeida et al., 2009) suggest we could expect high compliance, it is possible that our analysis with diurnal salivary cortisol variables are the subject of non-systematic noise from sampling error—a problem to which WAKING cortisol is particularly prone, given the complexities of the cortisol profile in the first hour post-waking (Fries et al., 2009). As such, interpretation of the diurnal cortisol samples, measured at home, should be interpreted advisedly. We did not take measures of head motion during the scan. However, we adjusted for this in the DT-MRI data, and visually examined tract, WMH and hippocampal segmentations (Section 2.5). Combined with the fact that participants in the current study are likely to represent the more motivated (agreeing to participant in an additional study component), probably healthier (based on the pre-selection criteria outlined in Section 2), and thus more scan-tolerant members of the cohort, will have greatly reduced the likelihood that movement artefacts contributed significant and non-trivial noise to our analyses. The temporal ordering of cognitive testing, cortisol sampling and MRI scanning was not optimal, spanning over a year in some cases. However, in the context of >60 years of cognitive change, between-subject differences in the lag between each stage of data collection are relatively small within a group with a very narrow age-range. Our sample is smaller than some other reports (e.g. Gerritsen et al., 2009, Comijs et al., 2010, Singh-Manoux et al., 2014; though they did not have MRI data), and the participants are generally healthy and are male, such that these results may not extrapolate to the general population. We also relied on commonly employed behavioural cut-offs and the absence of clinical diagnosis at initial recruitment to rule out pathological ageing as a potential driver of the reported effects (Section 2.1). The sample is also limited to healthy older males, precluding inference to older females. Although this design limits the generalizability of our findings, it has the advantage of excluding the potential confounding effect of age and gender.

It could also be argued that the number of comparisons undertaken requires Type I error correction. Table 2 illustrates the high degree of shared variance within measurement types (shaded cells; particularly so for cognitive and cortisol sampling measures), suggesting that correction may be overly conservative because each test is not an entirely independent opportunity for Type I error (Ott, 1999, Nyholt, 2001). Moreover, identifying 9 mediation candidates which satisfy the 3-way significance and directionality specified by the hypothesis (and that they are broadly consistent across two neuroradiologically distinct measures of white matter) is unlikely to be attributable to chance. Nevertheless, if our results are re-considered with a more stringent threshold of *p *< .01, no associations between brain and cortisol survive, leaving no candidates for mediation analysis and suggesting that these results should be interpreted with caution. Thus, in this exploratory analysis, it is possible that some of the reported findings are due to Type I error.

Finally, mediation analysis is asymmetric because a non-significant finding can be seen as model falsification, but a significant finding in mediation analysis only suggests support for the proposed hypothesis, though it may also be consistent with other models (Penke and Deary, 2010, Salthouse, 2011) and does not rule out alternative configurations of causation between these three variable types (so-called specification error). For example, in a simple mediation model, it is possible that all three variables are inter-correlated because they are reflections of a single common factor, or that there is an alternative causal direction. Thus, our findings could be explained because less cognitive decline reduces exposure to other factors that negatively affect WM and cognitive ability. Poorer WM integrity could then impair supra-HPA axis regulation during stressful situations, leading to elevated cortisol levels as a by-product. The current study lacks the power to model the subtle interplay among a large number of potentially confounding variables reliably (model parsimony is encouraged in small samples, e.g. Penke and Deary, 2010), and neither cortisol nor MRI measures were longitudinal. In future, well-powered longitudinal studies could examine cortisol–brain–cognition relationships in light of other factors which may affect brain ageing, such as diabetes (Di Dalmazi et al., 2012), vascular risk (Verhaaren et al., 2013), smoking and obesity (Debette et al., 2011) and physical activity (Gow et al., 2012) in order to ascertain more reliably the unique contribution that cortisol may make to brain and cognitive ageing.

In conclusion, the present study offers a novel perspective on covariances among cognitive change, cortisol levels and brain measures in older age. Results of a formal test of statistical mediation are in line with the hypothesis that brain WM structural integrity, but not hippocampal volume, mediates the relationship between elevated reactive cortisol and a relative decline in cognitive ability. Replications in larger cohorts which can more reliably detect smaller effect sizes are required to properly assess the unique contribution of cortisol to brain and cognitive ageing. The use of multi-modal MRI and focus on other potential cerebral sites beyond the hippocampus (Kremen et al., 2010b) and major WM tracts would also improve our understanding of the interplay between cortisol, the brain and cognition. The current findings may also be relevant to future studies in stress-related hypercortisolemic neuropsychiatric disorders.

## Conflict of interest

None.

## Funding

Direct financial support for this research was provided by Age UK and the cross council Lifelong Health and Wellbeing Initiative (MR/K026992/1). Brain imaging analysis was supported by the UK Medical Research Council (G0701120). The sponsors played no role in study design, collection, analysis and interpretation of these data, nor in the writing of the report and any decision to submit it for publication.

## Author contribution

The author contributions are as follows for each of the categories listed below:

Study conception & design, acquisition/analysis/interpretation of data: Cox, Macpherson, Ferguson, Royle, Muñoz Maniega, Bastin, MacLullich, Wardlaw, Deary.

Drafting the article or revising it critically for important intellectual content: Cox, Macpherson, Ferguson, Royle, Muñoz Maniega, Bastin, MacLullich, Wardlaw, Deary.

Final approval of the version to be submitted: Cox, Macpherson, Ferguson, Royle, Muñoz Maniega, Bastin, MacLullich, Wardlaw, Deary.

## Figures and Tables

**Table 1:** Descriptive statistics of study variables.

Variable	units	*n*	Mean	SD
WAKING	nmol/l	89	24	10.59
EVENING	nmol/l	84	3.47	2.75
DIURNAL	nmol/l	84	−20.77	9.72
START	nmol/l	86	16.39	7.77
END	nmol/l	89	12.67	6.07
REACTIVE	nmol/l	86	−3.87	7.19
MMSE	/30	90	28.54	1.52
WMH	mm^3^	89	12672.63	11037.29
HC	mm^3^	89	3228.05	369.24

WAKING: salivary cortisol levels taken at home on waking, EVENING: salivary cortisol levels taken at home at around 10 pm, DIURNAL: the slope (b–a) between WAKING and EVENING, START: salivary cortisol levels taken at the start of a cognitive testing appointment, END: salivary cortisol levels taken at the end of a cognitive testing appointment, REACTIVE: the slope (b–a) between START and END, MMSE: Mini Mental State Examination scores, WMH: white matter hyperintensity volume, HC: hippocampal volume.

**Table 2:** Correlations among cortisol, cognitive ageing differences and brain MRI variables.

		Salivary cortisol						Cognition				Brain MRI	
		Waking	Evening^a^	Diurnal	Start^a^	End^a^	Reactive	*g*_f_R	SpeedR	MemoryR	Age 11 IQ	*g*_MD_	WMH^b^
1	WAKING												
2	EVENING^a^	.21^c^											
3	DIURNAL	−.96^f^	.04										
4	START^a^	−.04	.11	.05									
5	END^a^	−.01	−.04	.09	.45^f^								
6	REACTIVE	.09	−.18	−.00	−.67^f^	.30^e^							
7	*g*fR	−.04	.05	−.02	-.28^d^	−.36^e^	.02						
8	SpeedR	−.06	.06	.02	−.36^e^	−.35^e^	.08	.72^f^					
9	MemoryR	−.00	−.07	−.07	−.35^e^	−.36^e^	.09	.68^f^	.53^f^				
10	Age 11 IQ	.22^d^	−.07	−.22^c^	.03	.08	−.06	.00	.00	.00			
11	*g*MD	.07	.23^c^	−.06	.25^d^	−.11	−.39^f^	−.25^d^	−.34^e^	−.28^d^	.07		
12	WMH^b^	.04	.04	.01	.24^d^	.21^d^	−.11	−.10	−.28^d^	−.25^d^	.14	.27^d^	
13	HC	.10	.20^c^	−.08	−.10	.05	.13	.35^e^	.24^d^	.23^d^	.19	.03	.12

Shaded cells illustrate correlations within measurement-type, i.e. cortisol, cognition, and brain imaging; clear cells denote tests driven by experimental hypotheses. WAKING: salivary cortisol levels taken at home on waking, EVENING: salivary cortisol levels taken at home at around 10 pm, DIURNAL: the slope (b–a) between WAKING and EVENING, START: salivary cortisol levels taken at the start of a cognitive testing appointment, END: salivary cortisol levels taken at the end of a cognitive testing appointment, *g*_f_: general cognitive ability, Speed: processing speed, Memory: memory ability, *R*: denotes that the variable was corrected for age 11 cognitive ability, *g*_MD_: general factor of tract mean diffusivity, WMH: white matter hyperintensity volume, HC: hippocampal volume. Tract–specific relationships with cortisol are reported in Cox et al., 2015.

a Log transformed.

b Square root transformed.

c Trend (*p *< .08).

d *p *< .05.

e *p *< .01.

f *p *< .001.

**Table 3:** Hierarchical linear regression between cognitive change and reactive cortisol measures.

	*β*1	*β*2	*F*	df	*R*^2^	*p*
*g*_f_						
+START	−.28		6.47	1, 76	.08	.013
+START + END	−.17^a^	−.26	6.37	2, 75	.13	.005

Speed						
+START	−.36		11.18	1, 76	.13	<.001
+START + END	−.27	−.21^b^	7.42	2, 75	.17	<.001

Memory						
+START	−.35		10.48	1, 76	.12	.002
+START + END	−.24	−.26	8.03	2, 75	.18	<.001

All predictors were significant (*p *< .05) apart from.

a *p *= .158.

b *p *= .073.

**Table 4:** Mediation Analysis.

			*β*		%	Mediation model		
*X*	*Y*	*M*	*c*	*c*’	Attenuation	*F* (df)	Lower CI	Upper CI
START^a^	*g*_f_R	gMD	−.34^d^	−.29^d^	**14.71**	**5.53 (2,67)**	**−.2573**	**−.0071**
START^a^	*g*_f_R	WMH^b^	−.28^c^	−.28^c^	0.00	3.20 (2,74)	−.0748	.1030
START^a^	SpeedR	gMD	−.42^e^	−.36^e^	**14.29**	**9.70 (2,67)**	**−.3147**	**−.0254**
START^a^	SpeedR	WMH^b^	−.35^d^	−.32^d^	**8.57**	**6.53 (2,74)**	**−.2665**	**−.0036**
START^a^	MemoryR	gMD	−.42^e^	−.36^d^	**14.29**	**8.40 (2,67)**	**−.3178**	**−.0047**
START^a^	MemoryR	WMH^b^	−.35^d^	−.31^d^	**11.43**	**6.67 (2,74)**	**−.2648**	**−.0005**
END^a^	*g*_f_R	WMH^b^	−.36^d^	−.35^d^	2.78	5.60 (2,77)	−.0890	.0562
END^a^	Speed*R*	WMH^b^	−.35^d^	−.31^d^	**11.43**	**7.08 (2,77)**	**−.2203**	**−.0074**
END^a^	Memory*R*	WMH^b^	−.36^d^	−.33^d^	**8.33**	**7.32 (2,76)**	**−.2286**	**−.0013**

*X*: independent variable, *Y*: outcome variable, *M*: mediator, *c*: path from *X* to *Y* (listwise, may differ slightly from pairwise associations reported in Table 2), *c*’: path from *X* to *Y* accounting for *M*. Bold type face indicates significant mediation effect (confidence intervals do not include 0; Preacher and Hayes, 2008). START: cortisol levels at the start of cognitive testing, END: cortisol levels at the end of cognitive testing, *g*_f_: general cognitive ability, speed: speed of processing, memory: memory ability, *R*: variable is a residual, corrected for age 11 cognitive ability. All tests of mediation are one-tailed and bias-corrected.

a Log transformed.

b Square root transformed.

c *p *< .05.

d *p* < .01.

e *p *< .001.
