# Supplementary figures and images for: Does white matter structure or hippocampal volume mediate associations between cortisol and cognitive ageing?
Source: Psychoneuroendocrinology. 2015 Dec;62:129–37. doi: 10.1016/j.psyneuen.2015.08.005 (PMC4642652; doi:10.1016/j.psyneuen.2015.08.005)

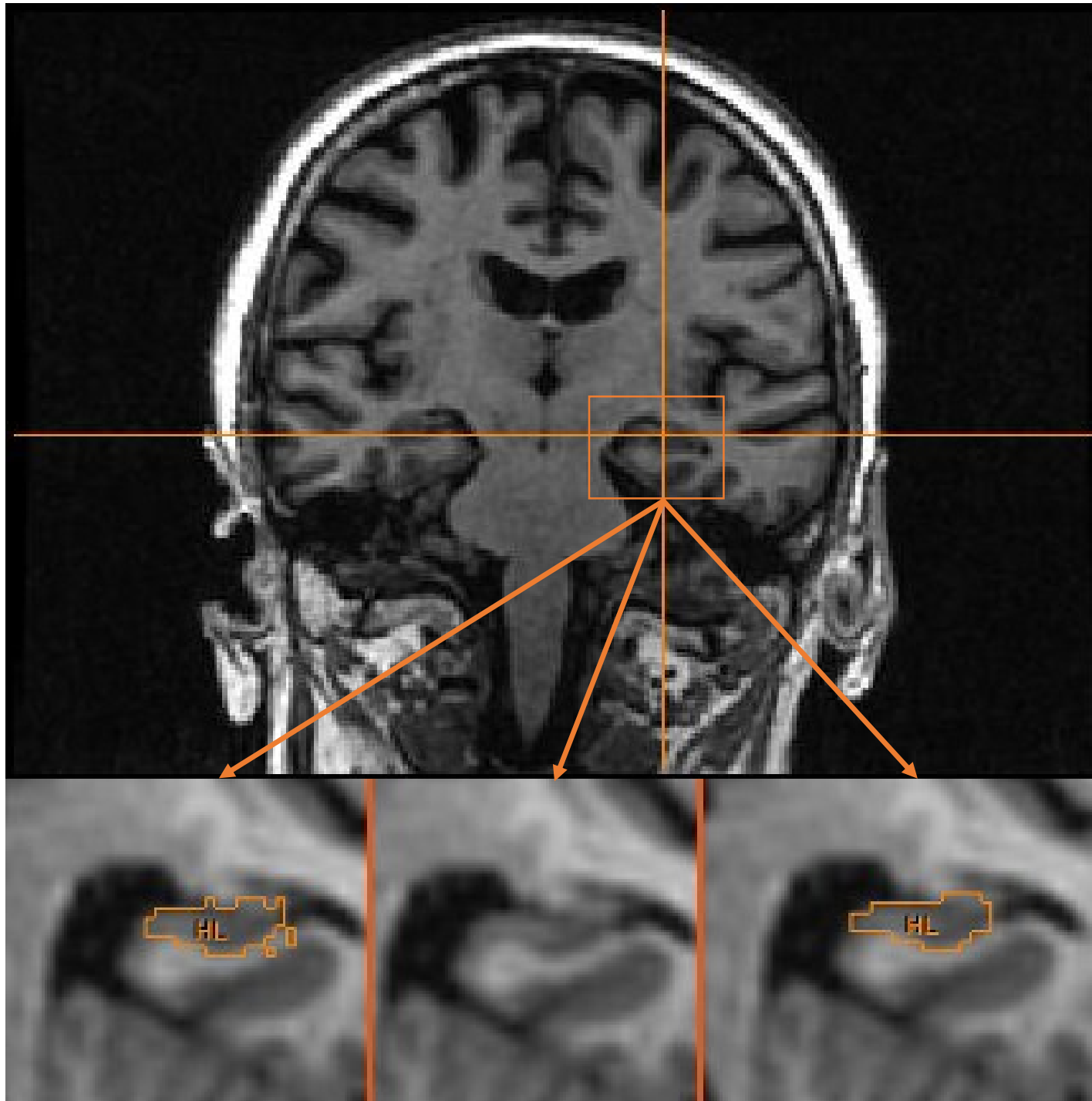

Supplement: Supplementary file 1 [file mmc1.pdf]
